# Unilateral congenital elongation of the cervical part of the internal carotid artery with kinking and looping: two case reports and review of the literature

**DOI:** 10.1186/1746-160X-3-29

**Published:** 2007-07-25

**Authors:** Nikolai A Ovchinnikov, Ramesh T Rao, Suresh R Rao

**Affiliations:** 1 Anatomy & Cell Biology Unit, Department of Preclinical Sciences, Faculty of Medical Sciences, University of the West Indies, St. Augustine, Trinidad and Tobago, West Indies; 2 Department of Paraclinical Sciences, School of Pharmacy, Faculty of Medical Sciences, University of the West Indies, St. Augustine, Trinidad and Tobago, West Indies

## Abstract

Unilateral and bilateral variation in the course and elongation of the cervical (extracranial) part of the internal carotid artery (ICA) leading to its tortuosity, kinking and coiling or looping is not a rare condition, which could be caused by both embryological and acquired factors. Patients with such variations may be asymptomatic in some cases; in others, they can develop cerebrovascular symptoms due to carotid stenosis affecting cerebral circulation. The risk of transient ischemic attacks in patients with carotid stenosis is high and its surgical correction is indicated for the prevention of ischemic stroke. Detection of developmental variations of the ICA and evaluation of its stenotic areas is very important for surgical interventions and involves specific diagnostic imaging techniques for vascular lesions including contrast arteriography, duplex ultrasonography and magnetic resonance angiography. Examination of obtained images in cases of unusual and complicated variations of vascular pattern of the ICA may lead to confusion in interpretation of data. Awareness about details and topographic anatomy of variations of the ICA may serve as a useful guide for both radiologists and vascular surgeons. It may help to prevent diagnostic errors, influence surgical tactics and interventional procedures and avoid complications during the head and neck surgery. Our present study was conducted with a purpose of updating data about developmental variations of the ICA. Dissections of the main neurovascular bundle of the head and neck were performed on a total 14 human adult cadavers (10 – Africans: 7 males & 3 females and 4 – East Indians: all males). Two cases of unilateral congenital elongation of the cervical part of the ICA with kinking and looping and carotid stenoses were found only in African males. Here we present their detailed case reports with review of the literature.

## Background

Among other vascular systems the system of carotid arteries represents a special interest for medical professionals involved in diagnosis and management of vascular diseases. High incidence of stroke, which is the third common cause of death in the United States, was associated by Faries et al [1] with high rate of carotid stenosis. Carotid occlusions could be caused by many factors including kinking and looping of the ICA. Such occlusions traditionally require surgical interventions with constantly developing techniques, which rely on the updated knowledge of many disciplines including developmental biology of vascular variations, both the congenital and acquired nature. Deviations of embryonic development of blood vessels from the most common patterns are frequently encountered and widely recognized [2-10]. A complicated process of transformation of the embryonic aortic arch system, which involves regression and disappearance, retention, or reappearance of its components, may result in congenital anatomical variations in the origin and courses of the vessels, Moore [7]. A differential growth may shift origin of some arteries, which then appear as anomalous.

Embryonic development of the carotid arteries is associated mainly with transformation of the first (the external carotid artery) and the third pair of the aortic arches. The common carotid artery is formed from the proximal part and the ICA from the distal part of the third aortic arches, Moore [7]. The latter joins the dorsal aorta. The ICA receives contributions from the upper intersegmental (proatlantal) and presegmental arteries, which connect it to the longitudinal neural (vertebral) artery and form carotid-vertebrobasilar anastomoses. Their persistence will result in development of anomalous branches of the ICA [5,10]. The cervical part of the ICA may give origin to the arteries, which usually originate from the ECA [3], and that may create complications during surgical interventions. Accidentally, the ICA may be absent on one or both sides of the neck [4]. A failure of embryonic absorption of the third aortic arch or the upper intersegmental artery may lead to congenital elongation of the ICA, Kelly [11] and its subsequent curving, kinking, tortuosity, looping.

In some cases the persistent carotid-vertebrobasilar anastomoses, shortening or elongation of the ICA with kinking and looping as well as its anomalous branches and variations in the course have no clinical significance, however, the knowledge of these variations might be useful and important for the interpretation of cranial contrast arteriography, MR angiography, duplex ultrasonography, because such variations can influence the surgical and other interventional procedures.

Here we present two specific cases of unilateral elongation of the cervical part of the ICA in African males showing kinking and looping with their detailed analysis and review of the literature.

## Materials and methods

Dissections of the main neurovascular bundle of the head and neck with a purpose of updating data about developmental variations of the ICA and preparation of the teaching and museum anatomical specimens were performed in the Gross Anatomy Laboratory on a total 14 human adult cadavers of both sexes 10 – Africans and 4 – East Indians. There were no any signs of trauma, surgery or wound scars of the neck in all cases. The skin of the head and neck, the superficial fascia of the neck, platysma muscles, and the superficial investing layer of the deep fascia of the neck were removed on both sides. Each parotid gland was removed by piecemeal along with its fascia and lymph nodes leaving terminal branches of the external carotid artery and accompanying veins intact. The sternocleidomastoid muscles were detached from the sternum and clavicle and shifted superoposteriorly. Both zygomatic arches were cut near their attachments to the skull and removed along with the masseter muscles. The base of the coronoid process of the mandibule was cut and shifted upward with the attachment of the tendon of the temporalis muscle. Each ramus of the mandible was separated from its body by a vertical osteotomy and removed after disarticulation of the mandibular head at the temporomandibular joint with attachments of both pterygoid muscles thus giving access to the content of the infratemporal fossa. The posterior belly of digastric, the styloid process with attached muscles and ligaments, the lateral and medial pterygoid muscles were removed on both sides in order to provide a better access to the highest part of the carotid sheath. The carotid sheath was traced within the carotid triangle on both sides from the root of the neck to the base of the skull. Its content including the common carotid artery (CCA), carotid bifurcation (CB), external carotid artery (ECA), carotid sinus (CS), internal carotid artery (ICA), vagus nerve (VN), internal jugular vein (IJV) and related structures were carefully dissected. Special attention was given to the course of the cervical part of the ICA. Measuring of the length and width of the ICA were conducted by a ruler. In two cases of anatomical variation of the cervical part of the ICA the midsagittal and coronal cross sections of the head and neck were made to facilitate a better approach to the site of anatomical variation of the ICA. In the first case the coronal cross section of the head and neck was produced in front of the anterior boundary of the foramen magnum just behind the pharyngeal tubercle of the occipital bone. In the second case the midsagittal cross section allowed examining the relationship of the ICA to the lateral pharyngeal wall, which was removed during the dissecting process. The ICA was traced along its entire course and its isolated specimen removed and studied. The size of the fourth part of the right and left vertebral arteries was compared. Distribution of the cadavers used in our study is shown in the Table 1.

**Table 1:** Distribution of cadavers used in this study.

**Origin**	**Sex**	**Age (years)**			**Number of cases**
	
		**50 – 59**	**60 – 69**	**70 – 79**	
**Africans**	Males **(Variations:)**	3 **(no)**	2 **(1 kinking of ICA)**	2 **(1 looping of ICA)**	7
	Females	1	1	1	3
**East Indians**	Males	0	2	2	4
	Females	0	0	0	0

				Total:	14

## Results

### Case Reports

#### Case 1

A 64-year-old, well built, tall (height 182 cm, weight 90 kg) African male cadaver showed a pronounced unilateral variation in the length (elongation) and course (kinking) of the highest portion of the cervical part of his left ICA. The history of this cadaver was not available, but during dissection of his abdominal cavity massive adhesions between the visceral peritoneal lining of the adjacent abdominal organs, between the visceral and parietal peritoneum and the perforated peptic ulcer of the duodenal bulb were found. These findings were interpreted as signs of the acute peritonitis – the most obvious cause for the death of this patient. No signs of any other diseases or pathological conditions were detected during the process of anatomical dissection of this cadaver.

The left ICA of this patient arose from the CB of the left CCA at the level of the middle third of C3 vertebra (symmetrical to the right ICA). The cervical part of the left ICA (diameter 8 mm) ascended 52 mm by a spiral course from its origin marked by the CS (width 16 mm) to the cranial base. Being posterolateral to the ECA, the ICA turned during its ascend first posteromedially then anteromedially toward the lateral pharyngeal wall and, when approaching the base of the skull in the area of the pharyngeal recess, anterolaterally. It came into the direct contact with the petrous part of the temporal bone just anteromedial to the base of the styloid process and it was located there between the jugular process of the occipital bone posteriorly and the lower edge of the tympanic part of the temporal bone anteriorly, occupying an area about 9 mm wide just laterally to the external opening of the carotid canal (CC).

**Figure 1:**
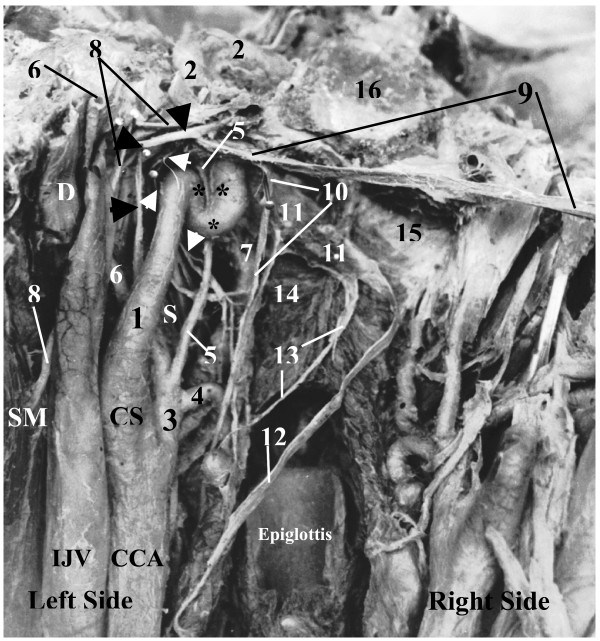
Posterior view of sharp kinking* "extracranial siphon" of the highest portion of the cervical part of the left internal carotid artery (ICA) on coronal cross section (upright position). **1 – **cervical part of ICA; **2 – **petrous part of ICA; **3 – **external carotid artery; **4 – **superior thyroid artery; **5 – **ascending pharyngeal artery; **6 – **occipital artery; **7 – **pharyngeal venous plexus; **8 – **accessory nerve (elevated); **9 – **vagus nerve (shifted to the right and upward); **10 – **superior laryngeal nerve; **11 – **superior cervical sympathetic ganglion (SCSG) (shifted to the right, forward and upward); **12 – **connecting trunk to middle cervical ganglion; **13 – **cardiac branch of SCSG; **14 – **superior pharyngeal constrictor; **15 – **pharyngo-basilar fascia; **16 – **basilar part of occipital bone; **CCA – **common carotid artery; **CS – **carotid sinus; **D – **posterior belly of digastric; **IJV – **internal jugular vein; **S – **stylopharyngeus; **SM – **sternocleidomastoid; **Black Arrowheads – **hypoglossal nerve (elevated); **White Arrowheads – **glossopharyngeal nerve (elevated)

**Figure 2:**
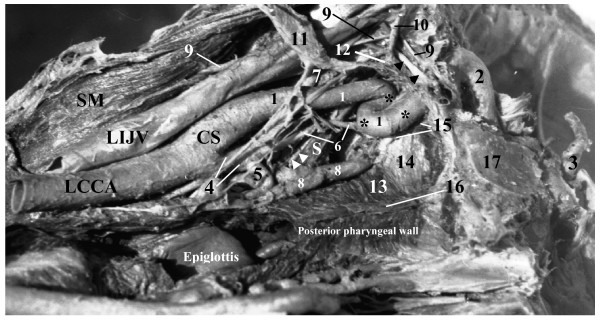
Posterior view of sharp kinking* "extracranial siphon" of the highest portion of the cervical part of the left internal carotid artery (ICA) on coronal cross section (horizontal position). **1 – **cervical part of ICA; **2 – **petrous part of ICA; **3 – **cerebral part of ICA; **4 – **external carotid artery; **5 – **superior thyroid artery; **6 – **ascending pharyngeal artery; **7 – **occipital artery; **8 – **pharyngeal venous plexus; **9 – **accessory nerve (elevated); **10 – **vagus nerve (shifted to the left and upward); **11 – **superior cervical sympathetic ganglion (shifted to the left and upward); **12 – **connection between superior cervical sympathetic ganglion and vagal ganglion; **13 – **superior pharyngeal constrictor; **14 – **pharyngo-basilar fascia; **15 – **lateral wall of pharyngeal recess; **16 – **posterior pharyngeal raphe; **17 – **basilar part of occipital bone; **LCCA – **left common carotid artery; **CS – **carotid sinus; **LIJV – **left internal jugular vein; **S – **stylopharyngeus; **SM – **sternocleidomastoid; **Black Arrowheads – **hypoglossal nerve; **White Arrowheads – **glossopharyngeal nerve

At the base of the skull the upper end of the ICA started a sinuous (kinking) course (Fig. 1) thus making the descending and ascending limbs of an extracranial siphon (ECS) of the ICA. From the inferior surface of the petrous bone the ICA sharply turned inferomedially and descended 13 mm along the medial surface of the upper end of this artery from the site of its curving. The site of the arterial bending was suspended to the base of the skull by a fibrous band, which had its ends attached to the inferior surface of the petrous bone just laterally to the fossula petrosa with the external opening of the canaliculus tympanicus. The fibrous band and petrous bone were forming a rigid fibroosseous ring through which the ICA was passing. There was a noticeable constriction of the ICA at this ring. The descending portion (limb) of the ECS of the ICA made a sharp bend turning medially and upward and ascended 13 mm as an ascending limb of the ECS toward the external opening of the CC. Because of anterolateral direction of the ICA approaching the external opening of the CC, it entered this canal obliquely making with the plane of the inferior surface of the cranial base a sharp angle open posteromedially. In the first portion of the CC the petrous part of the ICA ascended anterolaterally and then, after its sharp bend in the genu, it curved to become horizontal and pointed anteromedially towards the foramen lacerum (Fig. 2).

Both, the descending and ascending limbs of the ECS had rigid attachments to the petrous bone of the cranial base at two places, a kinking area was suspended by the fibrous band and the point of entry of the end of the cervical part of the ICA into the external opening of the CC also was attached to the rim of CC. Anteriorly, the ECS was related to the upper parts of the ascending pharyngeal artery and the pharyngeal venous plexus, the lateral end of the auditory tube and the tensor tympani muscle. Posterior to the ECS there were the upper part of the superior cervical sympathetic ganglion (SCSG), the glossopharyngeal nerve, the inferior vagal ganglion, the accessory nerve the hypoglossal nerve and the upper bulb of the IJV. The glossopharyngeal nerve had close relation and connection to the fibrous band, which suspended the curved (kinked) upper end of the ICA to the petrous bone. The inferior vagal ganglion was connected to the hypoglossal nerve. The SCSG had connections with the inferior ganglia of the glossopharyngeal and the VN and with the hypoglossal nerves.

The total length of all portions of the cervical (extracranial) part of the ICA constituted 78 mm. The course of the petrous, cavernous and cerebral parts of the left ICA was identical to the course of the right ICA and it corresponded to the common standard. The right ICA had its width at the beginning 16 mm, length of the cervical part 53 mm and diameter 8 mm.

**Figure 3:**
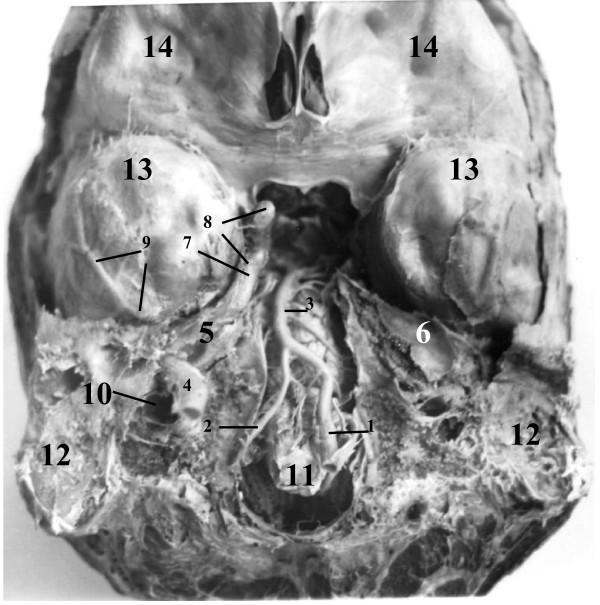
**Inferior view of the base of the brain removed from the cranial cavity with its roof and parts of the floor of the posterior cranial fossa**. **1 – **enlarged left vertebral artery (VA); **2 – **small right VA; **3 – **basilar artery; **4 – **upper end of cervical portion of right internal carotid artery (ICA); **5 – **inferior wall of right carotid canal (CC) in petrous bone; **6 – **superior wall of left CC in petrous bone; **7 – **part of right ICA corresponding to foramen lacerum; **8 – **cavernous part of right ICA; **9 – **right middle meningeal artery in endosteal layer of dura mater; **10 – **upper end of right internal jugular vein; **11 – **medulla oblongata; **12 – **basal part of mastoid process; **13 – **dura matter (DM) on inferior surfaces of temporal lobes; **14 – **DM on inferior surfaces of frontal lobes

Examination of the removed brain with its meninges revealed a significant asymmetry in the diameter of the fourth parts of the vertebral arteries (Fig. 3). The diameter of the left vertebral artery was 4.5 mm, which is almost in tree times more than the diameter of the right vertebral artery – 1.7 mm.

#### Case 2

A 70-year-old, African male cadaver (height 175 cm, weight 69 kg) showed another type of unilateral variation of the length (elongation) and course (looping) of the middle portion of the cervical part of his right ICA. The cause of death for this patient was an advanced prostate cancer with numerous retroperitoneal metastases in the region of the posterior abdominal wall. There was a significant hypertrophy of the urinary bladder (capacity of it was more than 1 liter) distention of both ureters and hydronephrosis of both kidneys with the renal failure. We did not notice any signs of other diseases or pathological conditions during the process of anatomical dissection of this cadaver.

**Figure 4:**
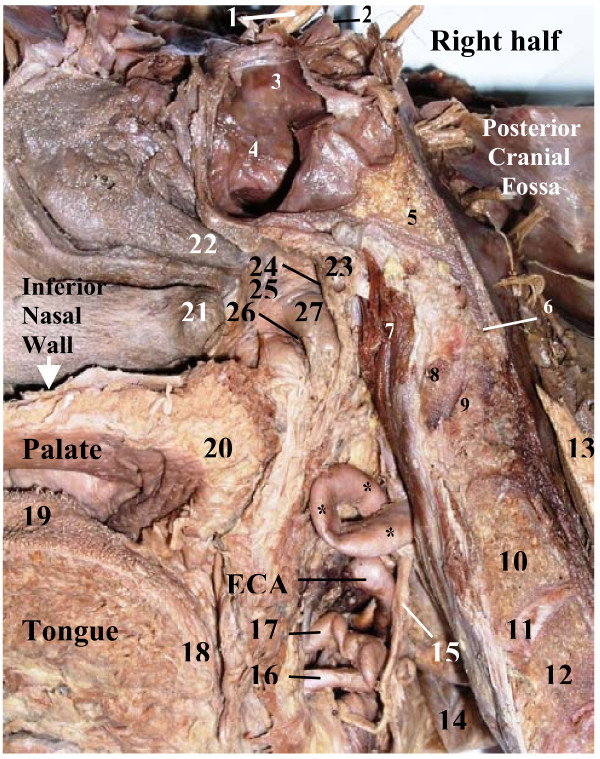
Medial view of looping* of the cervical part of the right internal carotid artery (ICA) on sagittal cross section of the head and neck (upright position). **1 – **optic nerve; **2 – **cerebral part of ICA; **3 – **cavernous part of ICA bulging wall of sphenoidal sinus (SS); **4 – **mucosal lining of right wall of SS; **5 – **basilar part of occipital bone; **6 – **anterior border of foramen magnum; **7 – **longus capitis; **8 – **anterior arch of atlas; **9 – **dens of axis; **10 – **body of axis; **11 – **intervertebral disk between C2 & C3 vertebrae; **12 – **body of C3 vertebra; **13 – **cervical part of spinal cord; **14 – **internal jugular vein; **15 – **superior laryngeal nerve; **16 – **lingual artery; **17 – **facial artery; **18 – **root of tongue (lingual tonsil); **19 – **dorsum of tongue; **20 – **soft palate; **21 – **posterior end of inferior nasal concha; **22 – **posterior end of middle nasal concha; **23 – **pharyngeal tonsil; **24 – **pharyngeal recess; **25 – **lateral wall of nasopharynx; **26 – **pharyngeal opening of auditory tube; **27 – **torus tubarius; **ECA – **external carotid artery

**Figure 5:**
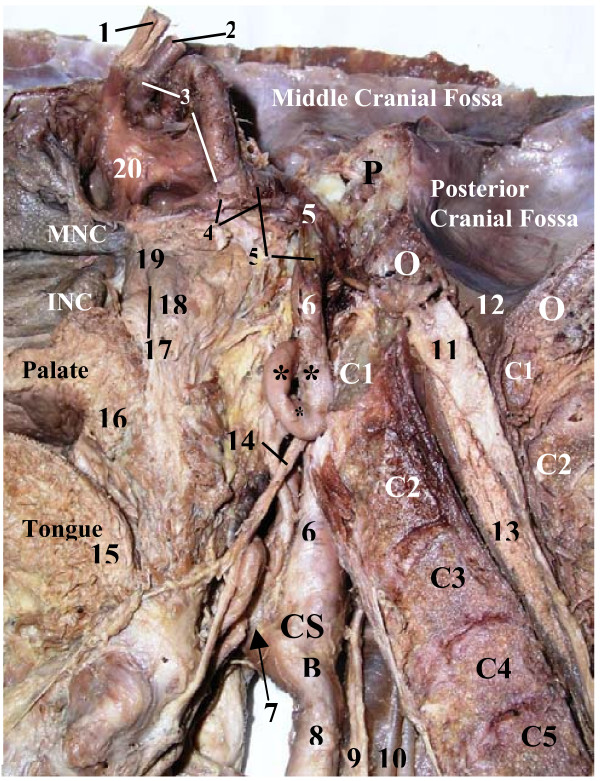
Medial view of looping* of the cervical part of the right internal carotid artery (ICA) on sagittal cross section of the head and neck (upright position). The medial parts of the first and second cervical vertebrae, basilar part of occipital bone, body of sphenoid bone and apical part of the pyramid of the temporal bone have been removed on right side to expose the entire course of the ICA. **1 – **optic nerve; **2 – **cerebral part of ICA; **3 – **cavernous part of ICA; **4 – **part of ICA overlying foramen lacerum; **5 – **petrous part of ICA; **6 – **cervical part of ICA; **7 – **external carotid artery; **8 – **common carotid artery; **9 – **vagus; **10 – **internal jugular vein; **11 – **superior cervical sympathetic ganglion (shifted medially and backward, overlying spinal cord); **12 – **lateral border of foramen magnum; **13 – **cervical part of spinal cord; **14 – **superior laryngeal nerve; **15 – **root of tongue (lingual tonsil); **16 – **soft palate; **17 – **pharyngeal opening of auditory tube; **18 – **torus tubarius; **19 – **lateral wall of nasopharynx; **20 – **mucosal lining of right wall of sphenoidal sinus; **B – **bifurcation of common carotid artery; **CS – **carotid sinus; **INC – **posterior end of inferior nasal concha; **MNC – **posterior end of middle nasal concha; **O – **occipital bone (cut surface); **P **– petrous part of temporal bone (cut surface); **C1, C2, C3, C4, C5 – **cervical vertebrae

The right ICA of this patient arose from the CB of the right CCA at the level of the middle third of C3 vertebra (symmetrical to the left ICA). The cervical part of the right ICA (diameter 6 mm) first ascended 40 mm from the site of bifurcation of the CCA (width of CS was 14 mm) in front of the transverse processes of C3, C2 and C1 vertebrae. At the level of C1 the ICA started a looping course (Fig. 4) where it sharply turned downwards making the upper bend and descended 22 mm along the anterior surface of the ICA. This part of the right ICA has formed a descending limb of its loop (Fig. 5). At the level of the upper border of the body of C2 vertebra the ICA sharply curved upwards making the lower bend and ascended 22 mm along the medial surface of the ICA thus forming an ascending limb of its loop. This limb crossed the level of the upper bend thus forming a complete loop and continued ascending 13 mm more upward and anterolaterally on the lateral wall of the pharyngeal recess toward the external opening of the CC, which it entered obliquely. Due to anterolateral direction of the ICA approaching the external opening of the CC, it entered this canal obliquely (similarly as in the first case) making with the plane of the inferior surface of the cranial base a sharp angle open posteromedially. The course of the petrous part in the CC was first upwards and anterolaterally and then, after its sharp bend in the genu, horizontally and anteromedially. The diameter of the fourth part of the right vertebral artery was about two times larger of its fellow on the left side.

**Figure 6:**
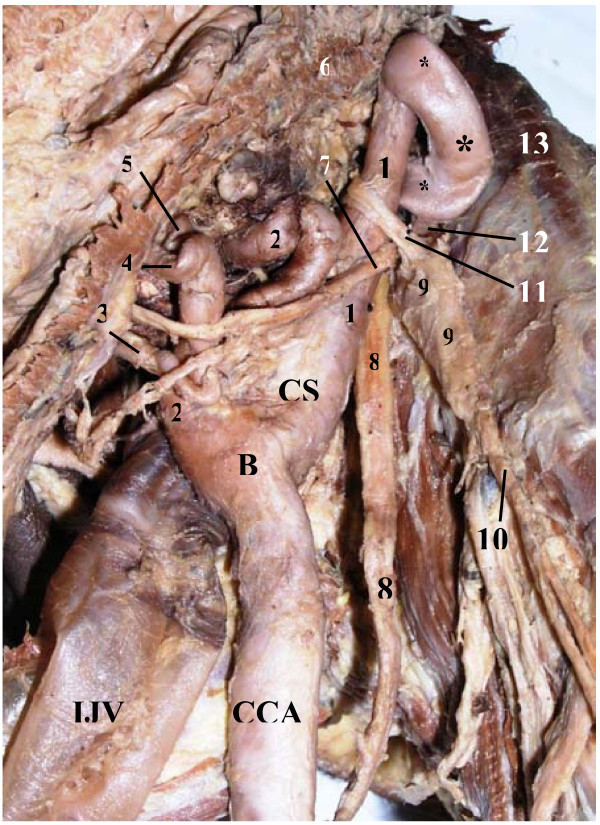
Medial view of looping* of the cervical part of the right internal carotid artery (ICA) on sagittal cross section of the head and neck (upright position). The pharynx has been shifted anteriorly for better view of relations. **1 – **cervical part of ICA; **2 – **external carotid artery; **3 – **superior thyroid artery; **4 – **lingual artery; **5 – **facial artery; **6 – **cut surface of pharyngeal wall; **7 – **superior laryngeal nerve; **8 – **vagus; **9 – **superior cervical sympathetic ganglion; **10 – **connecting trunk to middle cervical ganglion; **11 – **carotid nerve; **12 – **jugular nerve; **13 – **longus capitis; **B – **bifurcation of common carotid artery; **CCA – **common carotid artery; **CS – **carotid sinus; **IJV – **internal jugular vein

**Figure 7:**
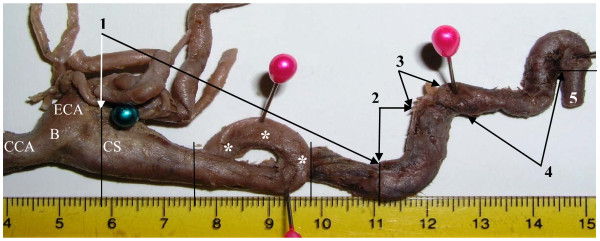
Medial view of the isolated specimen of the right carotid system of arteries with the looping* of the cervical part of the internal carotid artery (ICA). **Horizontal position**. **1 – **cervical part of ICA; **2 – **petrous part of ICA; **3 – **part of ICA overlying foramen lacerum; **4 – **cavernous part of ICA; **5 – **cerebral part of ICA; **B – **bifurcation of common carotid artery; **CCA – **common carotid artery; **CS – **carotid sinus; **ECA – **external carotid artery

The looping of the right ICA was located at the level C1 – C2 vertebrae anterolaterally to the longus capitis muscle. The VN and the IJV were located posterolateral to the loop. The latter was tightly packed within the carotid sheath and before its dissection the thickening of the carotid sheath was observed and interpreted as an arterial aneurism or a tumor. Even it resembled a large lymph node rather than the ICA itself. This thickening bulged the right wall of the nasopharynx inward and was located below and posteriorly to the nasopharyngeal opening of the auditory tube in the region of the palatopharyngeal sphincter (ridge of Passavant). The left wall of the nasopharynx did not have any bulging. The upper end of the right SCSG was located just posteroinferiorly to the lower bend of the loop (Fig. 6). The ICA below its loop was surrounded by two nerves the jugular and internal carotid originating from the upper end of this ganglion. The jugular nerve ascended to the cranial base on the posterior surface of the loop and on the highest portion of the cervical part of the ICA, whereas the internal carotid nerve crossed the artery medially and ascended on the anterolateral surface of the loop and on the upper portion of the ICA toward the CC. Anteriorly, this loop related to the connective tissue space containing the ascending pharyngeal artery, the pharyngeal venous plexus, the levator tympani and the lateral pterygoid muscles. The occipital artery, stylopharyngeus muscle and glossopharyngeal nerve related to the lateral aspect of the arterial loop. The isolated specimen of the right ICA with the loop, which shows all its parts and the ruler, is presented in Fig. 7.

The loop has significantly increased the total length of the cervical part of the right ICA, which constituted 97 mm. The course of the petrous, cavernous and cerebral parts of the right ICA was identical to the course of the left ICA and it corresponded to the common standard. The left ICA had its width at the beginning of the CS 14 mm, length of the cervical part 50 mm and diameter 6 mm. Apart from the above two cases of the unilateral elongation of the cervical part of the ICA its length in all other cadavers was more or less symmetrical on both sides ranging from 48 to 66 mm. The ICA ascended to the CC by a spiral course and was entering it obliquely in anterolateral direction forming with the plane of the cranial base an angle open posteromedially. The size of this angulation was a subject of individual fluctuation. The level of CB was mainly opposite the middle third of C3 vertebra. The size of the fourth part of the right and left vertebral arteries was also more or less identical.

## Discussion

The ICA is specified by Bannister et al [2] to be a major source of the arterial supply to the cerebral hemispheres. A classical description of the ICA given in Grays Anatomy indicates that it arises from the bifurcation of the common carotid artery lateral to the upper border of the thyroid cartilage level with the disc between the third and fourth cervical vertebrae. According to its course the ICA is subdivided into cervical, petrous, cavernous and cerebral parts. The cervical part ascends to the base of the skull within the carotid sheath in front of the transverse processes of the upper three cervical vertebrae to the external opening of the CC in the petrous temporal bone where it continuous with the petrous part of the ICA located within the CC. Leaving this canal via its internal opening the ICA overlies the cartilaginous plate of the foramen lacerum, ascends along the carotid grove of the sphenoid bone and enters the posterior end of the cavernous sinus, where it is known as the cavernous part of this artery. The ICA passes through this sinus from the posterior clinoid process to the anterior one, then makes a sharp bend superoposteriorly medial to the anterior clinoid process, forming its intracranial siphon, exits the sinus piercing its dural roof and continuous with the cerebral part of the ICA. Ziyal et al [12] proposed to subdivide this last part of the ICA into the clinoidal and cysternal segments based on its relations.

The posterolateral position of the ICA at its origin can be explained embryologically, since the ICA develops mainly from the dorsal aorta, which joins the third aortic arch, whereas the ECA arises from the ventral aorta Moore [7].

As it is indicated by Bannister et al [2] the length of the ICA artery varies with the length of the neck and the point of carotid bifurcation. Its cervical part is normally straight but on occasion may be very tortuous, being nearer to the pharynx than usual and very near the tonsil. Neither Bannister et al [2] nor Moore [7] and other standard anatomical texts discussed specific anatomical variations of the cervical part of the ICA, such as kinking, looping or coiling, associated with its elongation, though these variations are quite common and they have a great clinical significance[13].

Variations in the extracranial course of the ICA are not rare and they are frequently reported in the literature. Pharyngeal transposition of the ICA is considered to be a risk factor for acute hemorrhage in pharyngeal surgery [6,11,14-19]. Elongation of the ICA and associated curving, kinking, tortuosity, coiling, looping with their clinical significance are discussed by specialists [8,20-26]. Concerning its etiology, carotid elongation could have a congenital origin, or it might be an acquired condition [11,27-30]. The latter suggests that some pathological factors can trigger unproportional growth of the tunics of the ICA in which elongation of its adventitia would occur at a lesser extent then that of the muscular wall. That leads to the tendency to buckle and kink [13]. This process of elongation and kinking of the ICA thought to be exacerbated by atherosclerosis or fibromuscular displasia [8,31-33]. La Barbara et al [33] proposed a hypothesis in which the extracranial ICA is considered as a segment of transition between an elastic vessel (CCA) and a muscular vessel (intracranial ICA) and it is particularly a subject to metaplastic transformation, analogously to other transition zones in human body. Their results showed that elastic and muscular tissue of the ICA of their patients with kinking, coiling and tortuosity was substituted by loose connective tissue, configuring a metaplasia of its tunica media. The rarity of obstructive symptoms in patients before the age of 45 supports the acquired nature of kinking [13]. The curving of the ICA is mainly concerned with aging and coiling is usually ascribed to embryological causes [8]. Though kinking is bilateral in great majority of patients [13], we observed it only on one side. In our two cases of unilateral elongation of the ICA with its kinking (1^st ^case) and looping (2^nd ^case) the most obvious cause of elongation might have embryological nature. That is supported by presence of a firm attachment of kinking of the left ICA to the base of the skull by a fibrous band with a compensatory enlargement of the left vertebral artery in the 1^st ^case and a tight package of the looping of the right ICA within the carotid sheet and a compensatory enlargement of the right vertebral artery in the 2^nd ^case.

Occurrence rate of different forms of elongation of the ICA, which were reported in some countries, is shown in the Table 2. Almost all cases in this table pertain to Europeans, whereas our study used cadavers of African and East Indian origin.

**Table 2:** Incidence of elongation of the internal carotid artery and associated curving/kinking/tortuosity and coiling/looping reported in some countries.

**Authors**	**Year**	**Country**	**Number of all studied cases**	**Curving, Kinking, Tortuosity**		**Coiling, Looping**	
				
				**No of cases**	**%**	**No of cases**	**%**
Borioni et al [21]	1994	Italy	653	37	6.7	-	-
Koskas et al [32]	1993	France	2304	139	6.0	35	1.5
La Barbera et al [33]	2006	Italy	169	10	5.9	-	-
Pancera et al [29]	2000	Italy	590	368	62.4	-	-
Paulsen et al [8]	2000	Germany	282	86	30.5	5	1.8
Poulias et al [50]	1996	Greece	1123	38	3.4	21	1.9
Tillmann et al [18]	1995	Germany	89	4	4.5	-	-
Togay-Isikay et al [24]	2005	Turkey	345	78	22.6	7	2.0
Van Damme et al [26]	1996	Belgium	2188	62	2.8	-	-
Our study	2007	Trinidad	14	1	7.1	1	7.1

It was reported by Vannix et al in 1977 (cited from Alpagut et al [27]) that kinks are four times more common in women than in men. However, Paulsen et al [8] found no sexual differences and Koskas et al [32] observed 1.4 times male predominance. All these works were performed mainly on the white population. In our study both kinking and looping were present only in African males though the total number of cases studied was relatively small and number of African females was only 3 out of 14 cadavers. The vertebral level of carotid bifurcation in Africans was in general higher (middle of C3 vertebra) than the most common level for Europeans (disk between C3&4 vertebrae). Japanese individuals have their carotid bifurcation at the same level as Africans – lower third of C3 [34].

In normal condition tortuosity or a spiral course of an artery indicates on a high mobility of an area where it passes and it represents a compensatory mechanism in which a tortuous artery could be straightened during specific movements of mobile structures without being overstretched and/or occluded. For example, the tortuous facial artery will not be affected in wide opening of the mouth during depression of the mandible, the tortuous lingual artery will be straighten in protrusion of the tongue forward through the oral opening and tortuosity of the cervical part of the vertebral artery [35] is important for rotation of the head and atlas around the odontoid process of the axis.

The similar principle can be applied to the cervical part of the ICA, because movements of the head and neck may affect it. In all our cases the course of the cervical part of the ICA resembled a spiral course as it ascended. Having its position posterolaterally at the CB the ICA moved first posteromedially then anteromedially and when approaching the cranial base anterolaterally. The spiral course of the ICA could be clearly seen by using the Acland's Cross-Sectional Navigator [36] on the serial horizontal cross sections of the upper half of the neck. We did not find any comments in the literature we reviewed on this occasion and consider the spiral ascending pattern of the cervical part of the ICA as a compensatory mechanism during rotatory movements of the head and neck, which permits the ICA some degree of freedom during such movements and prevents it from overstretching and narrowing.

Elongation and tortuosity of the ICA is known for more than one century, but only in 1951 the association between carotid kinking and cerebrovascular insufficiency was made by Riser et al [37]. Since that report a number of authors have supported the clinical relationship of carotid elongation with cerebrovascular insufficiency [8,13,20,29,38]. A group of extracranial ICA anomalies, specifically kinking, tortuosity and coiling may cause symptomatic cerebrovascular insufficiency in 4–16% of the cases [33]. However, kinking without atheromatous plaque, even rather frequent, could be considered as a cause for carotid stenosis and stroke very rarely [13,32]. Pancera et al [29] also did not find any statistical correlation between kinking and stroke. Apart from presence of atheromatous plaque, the development of cerebrovascular symptoms depends upon the fortuitous positioning of the head and neck after some movements, when an elongated kinking ICA could be obstructed. This may affect the cerebral blood flow causing transient ischemic attack and even stroke [13].

The length of the left and right ICA in our reported cases was 78 and 53 mm (1^st ^case with kinking) and 50 and 97 (2^nd ^case with looping) respectively. We did not find any atheromatous plaque in both elongated arteries, but obvious indications on carotid stenosis. In the 1^st ^case the angle of kinking was close to 5 degrees (Fig. 1 &2), which belongs to "sharp kinking" or "Type 3" kinking according to classification suggested by Metz et al. [39]. The angle of kinking of the ICA was located in the fibroosseous foramen between the fibrous band suspending this artery and the petrous bone. The size of this foramen was less than diameter of the ICA passing through it, thus forming carotid stenosis. The ICA was unable to expand during its pulsation in that fibroosseous foramen, which had the rigid walls in its entire perimeter. In spite of the presence of carotid stenosis on the left side, the patient in the 1^st ^case seemed to be asymptomatic during his life. The deficiency of carotid circulation on the side of carotid stenosis could be compensated by his enlarged left vertebral artery (Fig. 3).

In the 2^nd ^case looping of the ICA was located 18 mm from the CB and its length was 22 mm. This correlates with data presented by Weibel et al [30]. The loop of the ICA was pressing on the lateral pharyngeal wall in the transitional area between the nasopharynx and oropharynx just superoposterior to the right tonsillar fossa and it was bulging this wall inwards. It could be a risk factor for acute hemorrhage in pharyngeal surgery. In the area of looping of the ICA the proximal and distal angles of looping were very sharp (as in the 1^st ^case) close to 5 degrees (Fig. 5). This also corresponds to "Type 3" kinking according to Metz's classification and indicates on a possibility of carotid stenosis. However, the presence of cerebrovascular symptoms in this patient during his life is not likely, because the deficiency of carotid circulation on the right side of the circle of Willis might be partly compensated via collateral root formed by the enlarged right vertebral artery as it was shown in the 1^st ^case.

An association of elongations of the ICA that is kinking, coiling, tortuosity and angulation with neurological symptoms and high stroke risk [20] supports surgical approach for correction of stenotic occlusions of the ICA in order to prevent stroke. Elongation of the cervical part of the ICA could be corrected by resection and removal of segments of the ICA [13] and connection of its cut ends by end-to-end anastomoses. It leads to shortening and straightening of the elongated ICA. It is our view that resection and removal of segments of the ICA or the CCA in patients with aneurysms [27,40] and other lesions [13] of the ICA without its prior elongation may result in shortening and overstretching of the ICA during movements of the head and neck. These may lead to its narrowing and cerebrovascular symptoms originating from the carotid stenosis. In addition, such stretching may affect vascular sutures at the end-to-end anastomoses and lead to their insufficiency or thrombosis [13]. Therefore, it is desirable, when necessary and possible, to apply specific techniques of elongation of arteries in patients before resection and removal of their arterial segments.

Carotid stenosis is traditionally treated by carotid endarterectomy [1]. The letter depends on proper preoperative studies of the patients, which include contrast arteriography [41], duplex ultrasonography [21,29] and MR angiography [9,25]. Over recent years new effective endovascular techniques including carotid angioplasty and stenting have been developed [42,43] to prevent stroke in patients with carotid artery occlusive disease. Indications for the surgical correction of carotid stenosis are based on the degree of vascular occlusions, which require proper measuring of occlusion sites of the ICA [41] by special techniques. Surgical skills with a low complication rate are absolutely essential for correction of carotid occlusions [34], which also require a sound knowledge of the topographic anatomy of the neck. During surgical procedures in the neck, carotid arteries and branches of the ECA are considered as important landmarks Ord et al [44]. The arteries of the neck should be identified before cross-clamps are placed and arteriotomy is performed, because they may have unusual origin.

Kinking of the highest portion of the cervical part of the ICA with carotid stenosis may require its surgical correction too [19,20,31,34,45-47]. This portion of the ICA could also be injured in motor vehicular accidents. However, surgical treatment of lesions of this part of the ICA is exceptionally challenging, because an exposure of its para-mandibular and para-atlantoaxial segments is limited [48,49]. The surgical access to these segments is insufficient for manipulations needed for optimal repair due to the bony interference of the angle of the mandible and the mastoid process. Various techniques of approach to the cranial base usually involve significant surgical morbidity, when important anatomical structures are sacrificed. In several reports authors [40,46,49,50] advocate radical mastoidectomy via preauricular and posterior auricular incisions in addition to the incision described by DePalma [45] (an incision from behind the ear lobe into the neck lines inferiorly). The removal of a portion of the mastoid bone and release of the facial nerve to the stylomastoid foramen allow distal exposure of the ICA in the petrous portion of the temporal bone. This technique, however, is associated with facial nerve paralysis and sacrifice of middle ear function. In comparison with the above techniques, mandibular subluxation (distraction) and mandibular vertical ramus osteotomy have been associated with low rates of surgical morbidity [48]. These two techniques also can be accomplished with little additional surgical time. In our 1^st ^case the fibrous band firmly attached kinking of the ICA to the base of the skull and this could make a surgical repair of kinking even more complicated due to its close relationship to the last four cranial nerves, SCSG, IGV and ascending pharyngeal artery. In addition, due to close approximation of the fibrous band to the glossopharyngeal nerve and to the fossula petrosa it could be a possibility that the tympanic branch of the IX CN was passing through this fibrous band and its division would sacrifice the preganglionic parasympathetic fibres for the parotid gland. Endovascular techniques such as carotid angioplasty and stenting, designed for dilation of stenotic areas would not work in this case, because of a rigidity of the fibroosseous ring that narrowed the ICA.

In our study we found that the first portion of the petrous part of the ICA in the CC ran upwards and anterolaterally having the same direction as the highest portion of the cervical part of the ICA, which entered the CC not perpendicular to of the base of the skull, but obliquely making with the horizontal plane of the cranial base an angle opened posteromedially. The ascending portion of the petrous part of the ICA turned within a genu of the CC almost under the right angle to be continuous with the horizontal portion and passed anteromedially. Oblique anterolateral course of the ascending portion of the petrous part of the ICA and sharp bend in the genu of the CC may be associated with an increased pressure of the pulsating artery on the lateral wall of the CC in the area of its genu. The lateral wall of the CC was rather thin in our cases. We suggest a hypothesis that in some individuals, whose posteromedial angle of entry of the ICA into the CC might be rather sharp, the pressure of this artery on the lateral wall of the CC would be higher and that could lead to atrophy of the bony tissue in some circumstances and complete disappearance of the lateral wall of the CC with a subsequent formation of the aberrant ICA located in the middle ear cavity. It is a well known fact that a structure pressing on the bone forms a depression on the bone surface due to resorption of bone tissue by the osteoclasts. This approach may help to understand an uncertain cause observed by Caldemeyer et al [5] of an aberrant ICA passing through the middle ear cavity due to an absence of the posterolateral wall of the CC. Further studies of hydrodynamics of the ICA and morphofunctional properties of the bone tissue are required to confirm this hypothesis.

## Conclusion

Two cases of unilateral elongation of the cervical part of the ICA with kinking and looping and obvious carotid stenosis in African males, presented in our study, have clearly demonstrated some morphological details, which could not be obtained during clinical examination of patients involving modern imaging techniques. A possibility of insufficient cerebrovascular circulation due to carotid stenosis in both cases could be compensated by the enlarged vertebral arteries on the sides of carotid occlusions, which may suggest their congenital nature. Correct interpretations of diagnostic images obtained in cases of unusual and complicated variations of vascular pattern of the ICA require awareness about them in addition to sound knowledge of developmental and topographic anatomy. Comprehensive professional understanding of causes of elongation of the ICA leading to carotid stenoses may help to develop new and more effective techniques of their correction. Information about details and topographic anatomy of the reported and other variations of the ICA may serve as a useful guide for both radiologists and vascular surgeons. It can help to prevent diagnostic errors, influence surgical and interventional procedures and avoid surgical complications during head and neck surgery. Variations of the ICA may be asymptomatic, so an extra care must be undertaken even during routine surgical interventions involving tonsillectomies, removal of adenoids and other head and neck surgeries.

## Competing interests

The author(s) declare that they have no competing interests.

## Authors' contributions

NAO has made substantial contributions to conception, design, dissections, acquisition, analysis and clinical interpretation of data, review of the literature, acquisition and processing of all illustrative materials, preparation of case reports and tables; drafting and revising critically the second version of the manuscript and its final proofing, alignment, formatting and completion of final version for publication

RTR participated in the design of the study, dissections, acquisition, analysis and interpretation of data, review of the literature, drafting the first version of the manuscript, assisted with revising of its second and final versions

SRR participated in the design of the study, dissections, acquisition, analysis and interpretation of data, review of the literature, drafting the first version and revising the second and final version of the manuscript and he communicated with the BioMed Central Editorial Production Team
